# Supplementary material for: Machine learning-enabled constrained multi-objective design of architected materials
Source: Nat Commun. 2023 Oct 19;14:6630. doi: 10.1038/s41467-023-42415-y (PMC10587057; doi:10.1038/s41467-023-42415-y)
Supplement: Supplementary file 1 — Supplementary information [file 41467_2023_42415_MOESM1_ESM.pdf]

Supplementary Information for

Machine learning-enabled constrained multi-objective design of architected materials

Bo Peng#, Ye Wei#*, Yu Qin#*, Jiabao Dai, Yue Li, Aobo Liu, Yun Tian, Liuliu Han,

Yufeng Zheng, Peng Wen*

*Corresponding authors: Ye Wei (ye.wei@rwth-aachen.de), Yu Qin (qinyu95@126.com), Peng Wen (wenpeng@tsinghua.edu.cn)

The PDF file includes:

Supplementary Figures **2**
Supplementary Figure 1: Representation of the constrained multi-objective optimization task in this study. 2
Supplementary Figure 2: Porosity-modulus relation of Ti scaffolds 3
Supplementary Figure 3: Topology optimization of porous bone implant by tuning porosity. 4
Supplementary Figure 4: Performance evaluation of the 3D-CNNs on the Ti test dataset. 5
Supplementary Figure 5: Performance evaluation of the 3D-CNNs on the Zn testing dataset. 6
Supplementary Figure 6: Training of the 3D-CAE. 7
Supplementary Figure 7: The average negative log-likelihood versus the number of clusters in the GMM. 8
Supplementary Figure 8: Micro-computed tomography (Micro-CT) and 3D model of the Ti cubic scaffolds. 9
Supplementary Figure 9: The performance comparison on the Rosenbrock optimization problem. 10
Supplementary Figure 10: The overall data distribution of Ti finite element simulation results. 11
Supplementary Figure 11: Compression test curves of the Ti cubic scaffolds. 12
Supplementary Figure 12: Regression activation map of the Ti cubic scaffolds. 13
Supplementary Figure 13: The porosity matrices and ‘face-centered’ lattice structures of the Ti cubic scaffolds. 14
Supplementary Figure 14: FEM analysis of the Ti cubic scaffolds. 15
Supplementary Figure 15: The overall data distribution of Zn finite element simulation results. 16
Supplementary Figure 16: Characterization of the Zn cubic scaffolds. 17
Supplementary Figure 17: Experimental strain–stress curves of the Zn cubic scaffolds. 18
Supplementary Figure 18: FEM analysis of the Zn cubic scaffolds. 19
Supplementary Figure 19: Pipeline for irregular-shaped scaffold design. 20
Supplementary Figure 20: Experimental displacement-force curves of bone implants. 21
Supplementary Figure 21: FEM analysis of the anatomic bone implants. 22
Supplementary Figure 22: The schematic for the smooth transition between units with different porosities. 23
Supplementary Figure 23: Powder observation. 24
Supplementary Figure 24: Laser powder bed fusion machines of the Ti and Zn cubic scaffolds. 25
Supplementary Figure 25: Cross-section images of the Ti and Zn cubic scaffolds 26
Supplementary Figure 26: Experimental strain–stress curves of solid Ti and Zn. 27
Supplementary Figure 27: The camera pictured Ti cubic scaffolds. 28
Supplementary Figure 28: Random-search-based active learning. 29
Supplementary Figure 29: Bayesian-optimization-based active learning. 30
Supplementary Figure 30: Finite element simulation results comparison of newly prepared initial dataset 31
Supplementary Tables **32**
Supplementary Table 1: The Ti cubic scaffolds - Mean and standard deviation of the elastic modulus and yield strength at each learning iteration. 32
Supplementary Table 2: Experimental results of ML design and uniform design Ti cubic scaffolds. 33
Supplementary Table 3: The Zn cubic scaffolds - Mean and standard deviation of the elastic modulus and yield strength at each learning iteration. 34
Supplementary Table 4: Experimental results of ML design and uniform design Zn cubic scaffolds. 35
Supplementary Table 5: The actual porosity of scaffolds from Micro-CT characterization. 36
Supplementary Table 6: Comparison of GAD-MALL, random-search-based active learning and Bayesian-optimization-based active learning. 37
Supplementary Methods **38**
Supplementary References **39**

Supplementary Figures

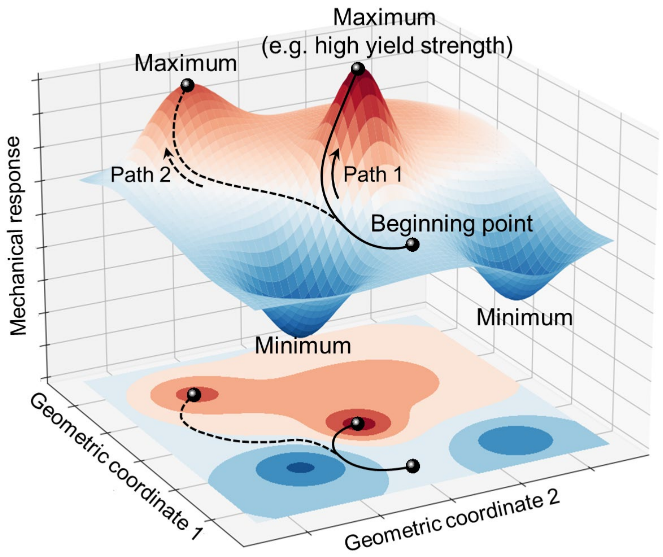

Supplementary Figure 1: Representation of the constrained multi-objective optimization task in this study. The task in this study can be mathematically formulated as follows:

$$\text{Find } x \in H$$
$$\text{To the mapping } f : H \rightarrow Y \text{ and } g : H \rightarrow E$$
$$\text{Such that } x = argmax_{x \in H}\left(f\left(x\right)\right) \text{ and } g\left(x\right) = E_{target}$$
$$\text{Under the constraint: weight} <= \text{fixed constant}$$

H is the scaffold design space; f and g are the mappings of scaffold design to its corresponding yield strength (Y) and elastic modulus (E). The contour surface of this figure represents the arrangement with equal E. The task is to find the maximum yield point on this surface.

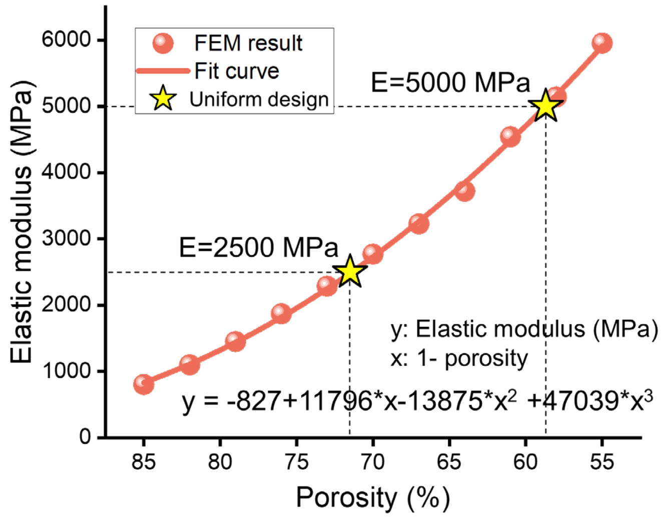

Supplementary Figure 2: Porosity-modulus relation of Ti scaffolds.

The protocol to find the uniform design with the desired elastic modulus: 1) Perform finite element simulations to 11 uniform designs with different porosities vary from 55% to 85%. 2) Plot the porosity-modulus points and fit a curve along the points. 3) Determine the corresponding porosity of a specific design by substituting the desirable modulus into the fitted porosity-modulus relation. Source data are provided as a Source Data file.

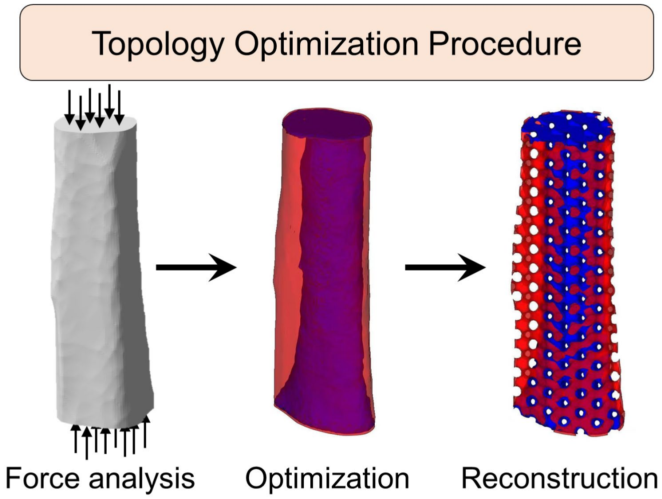

Supplementary Figure 3: Topology optimization of porous bone implant by tuning porosity.

We tried the "Shape-retained FGCS optimization" method from the reference[1] and achieved to match the elastic modulus by adjusting the porosity in the "retain"(blue) and "remove"(red) spaces, as shown in the figure below.

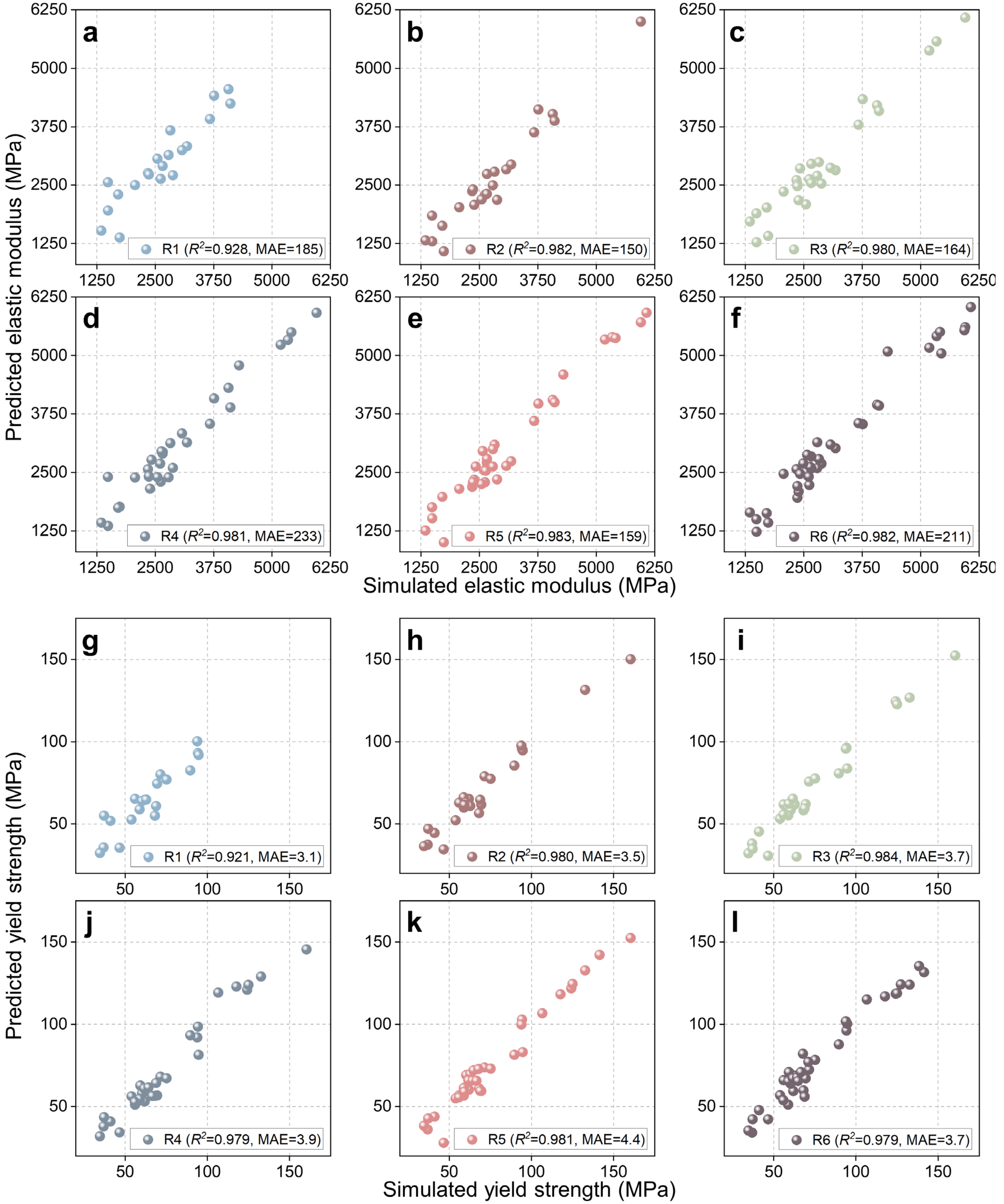

Supplementary Figure 4: Performance evaluation of the 3D-CNNs on the Ti test dataset.

The coefficient of determination (R^2) and mean absolute error (MAE) of the 3D convolutional neural networks (3D-CNNs) from rounds 1 to 6, **a**, **b**, **c**, **d**, **e**, **f** refer to the E and **g**, **h**, **i**, **j**, **k**, **l** refer to the Y. All 3D-CNNs showed high accuracy in the regression tasks (R^2 ratio > 0.97) at each active learning iteration except the round 1. The test dataset was uniformly sampled from the labeled dataset. Source data are provided as a Source Data file.

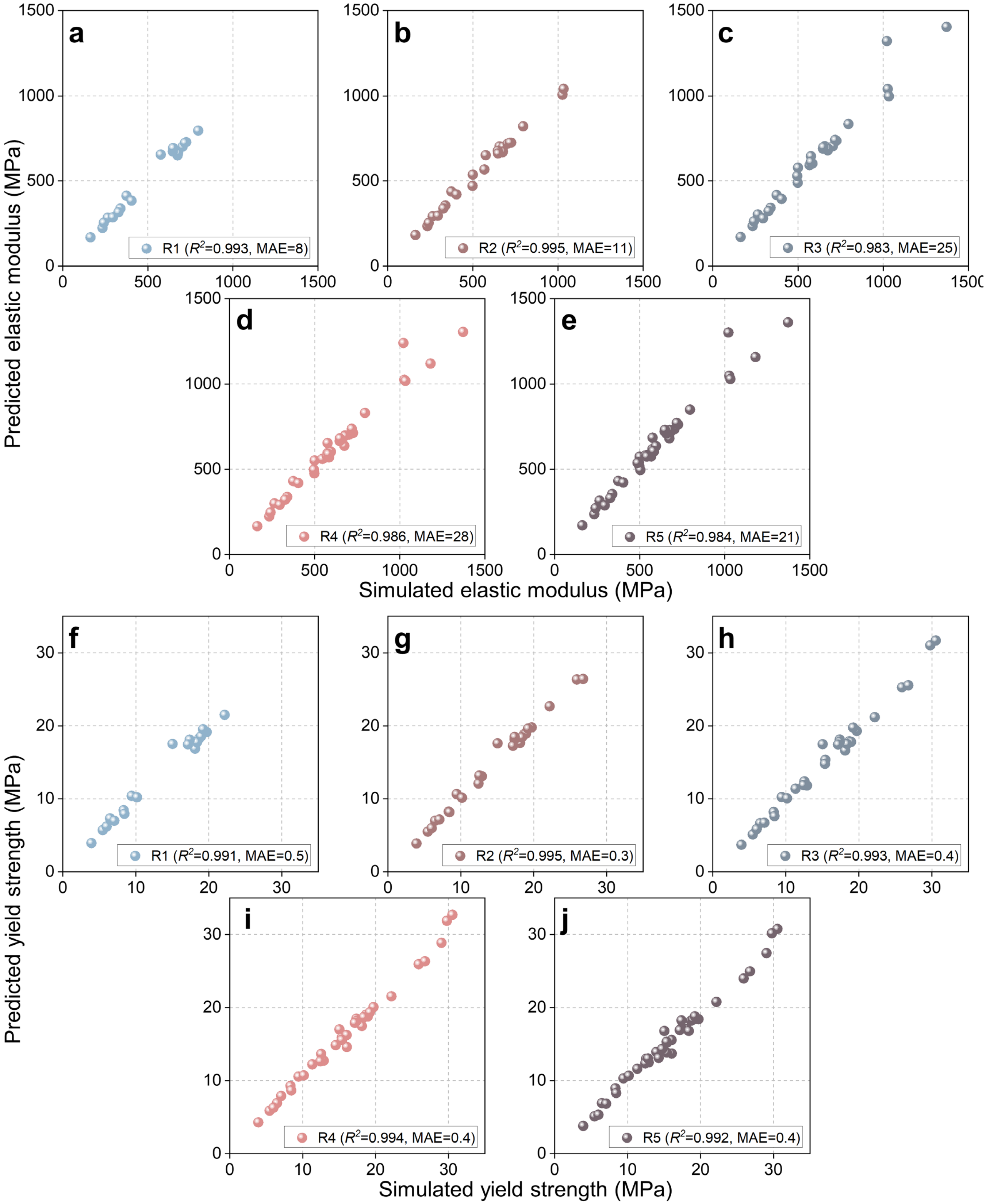

Supplementary Figure 5: Performance evaluation of the 3D-CNNs on the Zn testing dataset.

The coefficient of determination (R^2) and mean absolute error (MAE) of the 3D convolutional neural networks (3D-CNNs) from rounds 1 to 5, **a**, **b**, **c**, **d**, **e** refer to the E and **f**, **g**, **h**, **i**, **j** refer to the Y. All 3D-CNNs showed high accuracy in the regression tasks (R^2 ratio > 0.98) at each active learning iteration. The test dataset was uniformly sampled from the labeled dataset. Source data are provided as a Source Data file.

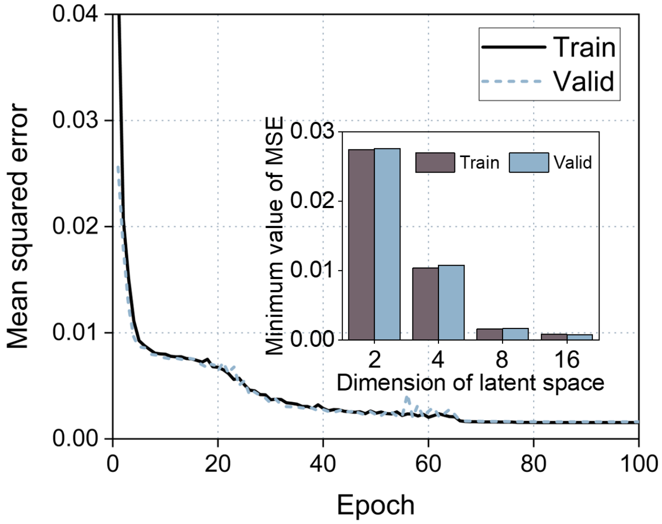

Supplementary Figure 6: Training of the 3D-CAE.

The training history of the 3D convolutional autoencoder (3D-CAE) with eight latent dimensions. The loss quickly dropped to near zero after 60 epochs. The histogram (inset) shows that the loss of 4-dimensional latent space was high, while sampling from the 16-dimensional space was time-consuming. The use of 8 dimensions reached a balance between loss and efficiency. We performed the hyperparameter search using the Bayesian hyperparameter optimization and the goal is to minimize the reconstruction loss. We tune the filter size, kernel size and the number of layers. Source data are provided as a Source Data file.

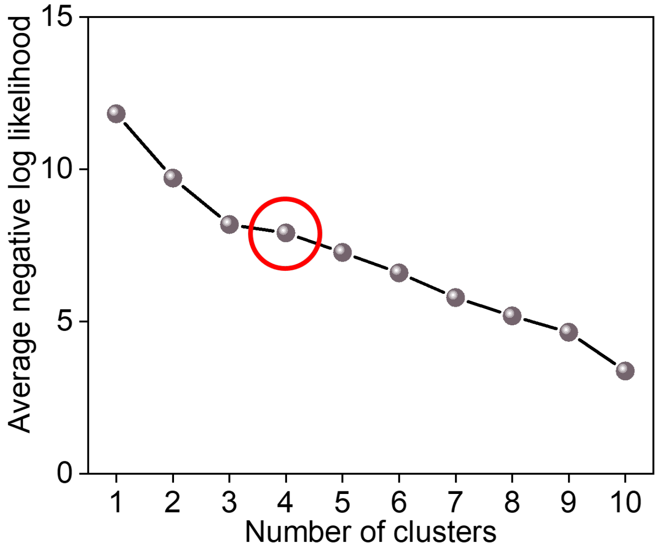

Supplementary Figure 7: The average negative log-likelihood versus the number of clusters in the GMM. The Gaussian mixture model (GMM) was used to estimate the density in the latent z space (i.e., the marginal posterior $q_{\varphi}(z)$). The GMM is a density estimation model that uses a mixture of a finite number of Gaussian distributions with unknown mean and covariance to fit the data points. The number of Gaussian distributions is usually determined via the empirical elbow method. The elbow method is a heuristic used in determining the Pareto fronts in multi-objective optimization; in this case, it was used to determine the potential optimal number of clusters. As shown in the figure, the average negative log-likelihood was plotted as a function of the number of clusters, and we selected 4 as it represents the 'elbow' of the curve. Source data are provided as a Source Data file.

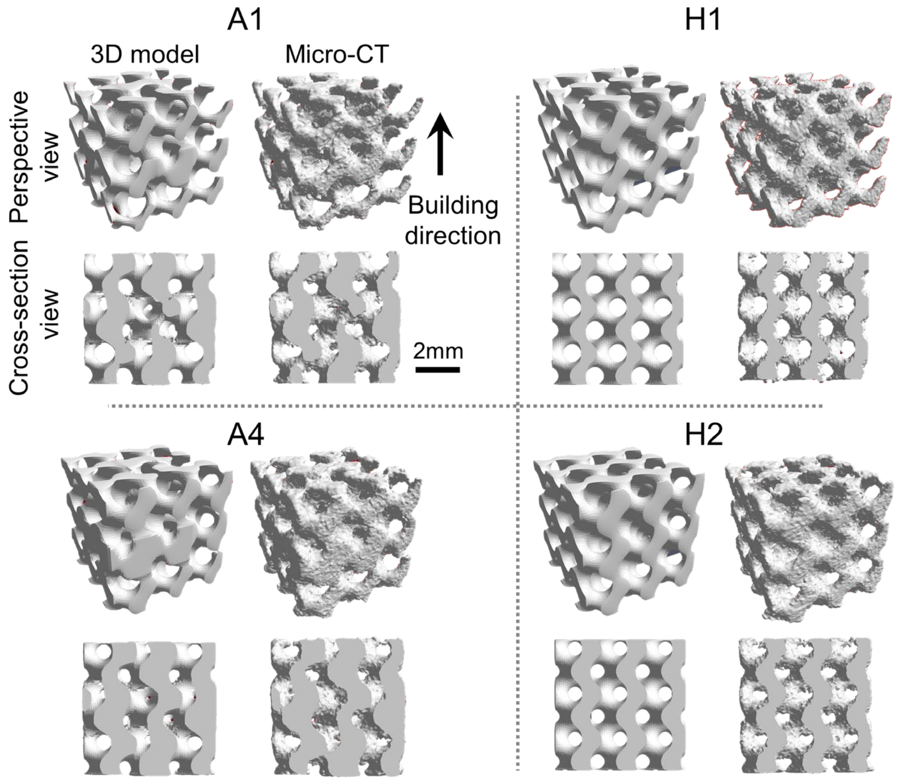

Supplementary Figure 8: Micro-computed tomography (Micro-CT) and 3D model of the Ti cubic scaffolds. Machine learning (ML) designs (A1 and A4) and uniform designs (H1 and H2).

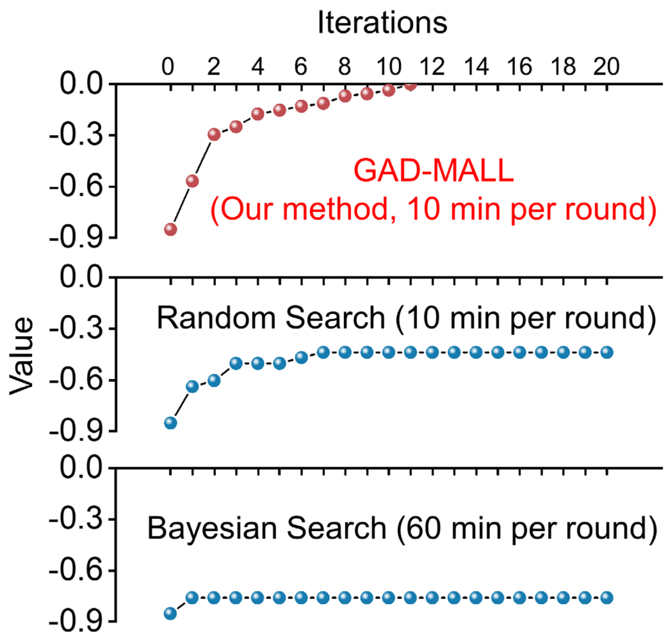

Supplementary Figure 9: The performance comparison on the Rosenbrock optimization problem. The sign was reversed since the algorithm was designed for the global maximum search. It is obvious that the GAD-MALL outperform other two methods, reaching global maximum with only 10 rounds, whereas Random search and Bayesian search reaches plateau after first few rounds. More details see Supplementary Methods. Source data are provided as a Source Data file.

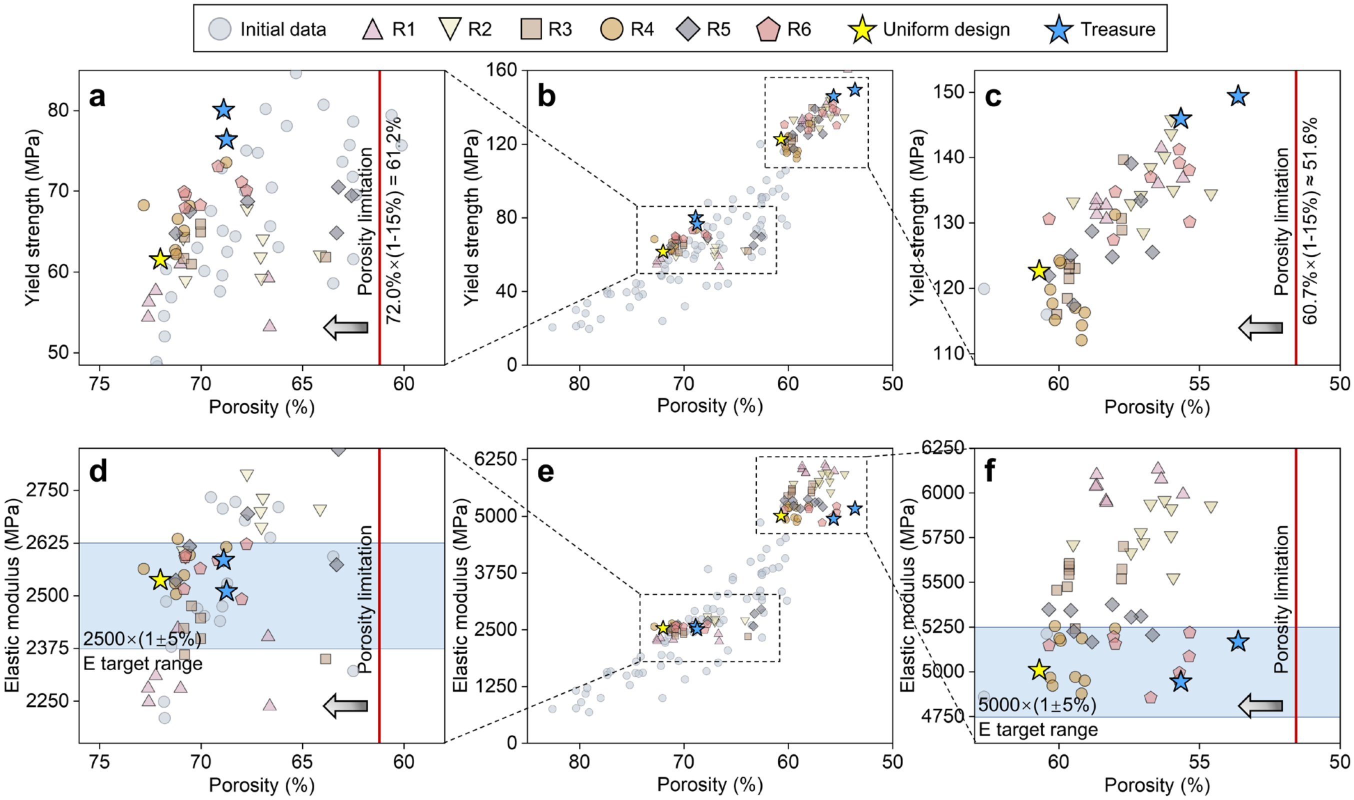

Supplementary Figure 10: The overall data distribution of Ti finite element simulation results.

a, **b**, **c** The porosity-yield strength (Y) plot. The red line indicates the porosity limitation. **d**, **e**, **f** The overall data distribution in terms of the porosity-elastic modulus (E) plot. Initial data points are marked as light blue dots. Six rounds of active learning data points are represented by various other colored symbols (R1-R6). Uniform design and GAD-MALL treasure points are marked as golden and blue stars, respectively, for easier comparison. The blue shaded area indicates the target range of E (i.e., target E value ± 5%). Source data are provided as a Source Data file.

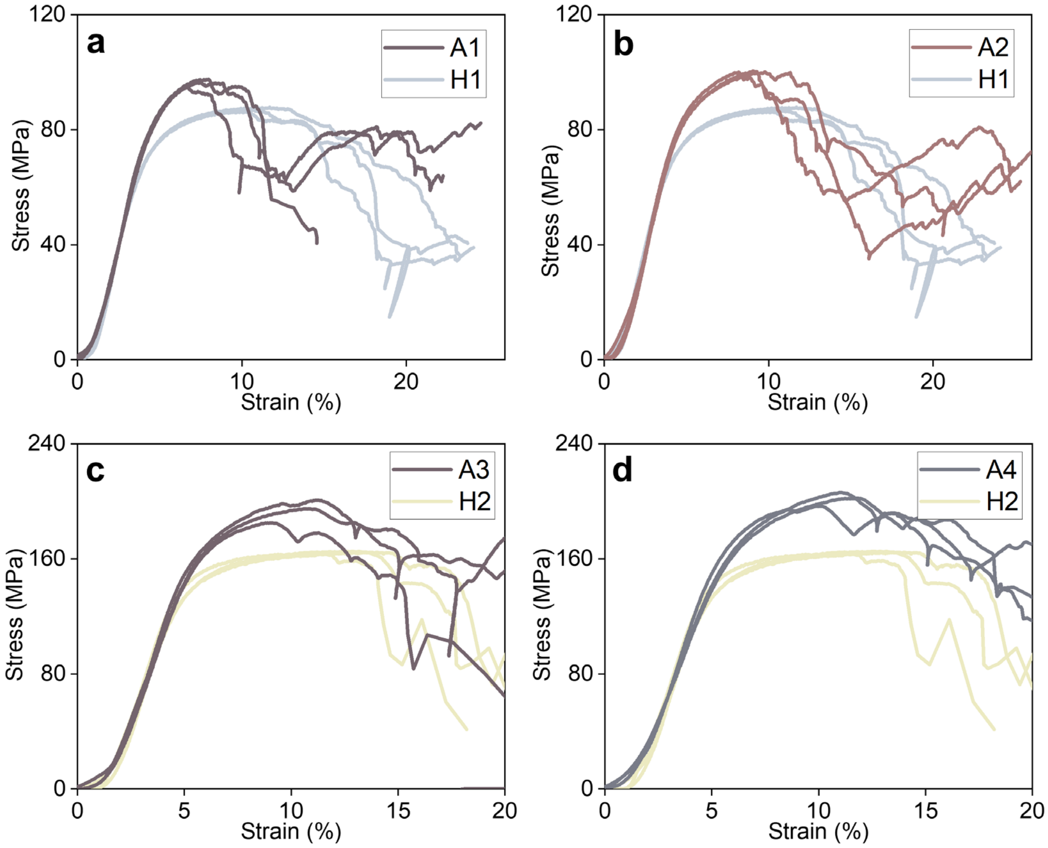

Supplementary Figure 11: Compression test curves of the Ti cubic scaffolds.

Machine learning (ML) designs (A1-A4) and uniform designs (H1 and H2). Three replicates were tested to ensure reproducibility. In each figure, all stress-strain curves are adjusted in the x-axis direction to make them overlap. Source data are provided as a Source Data file.

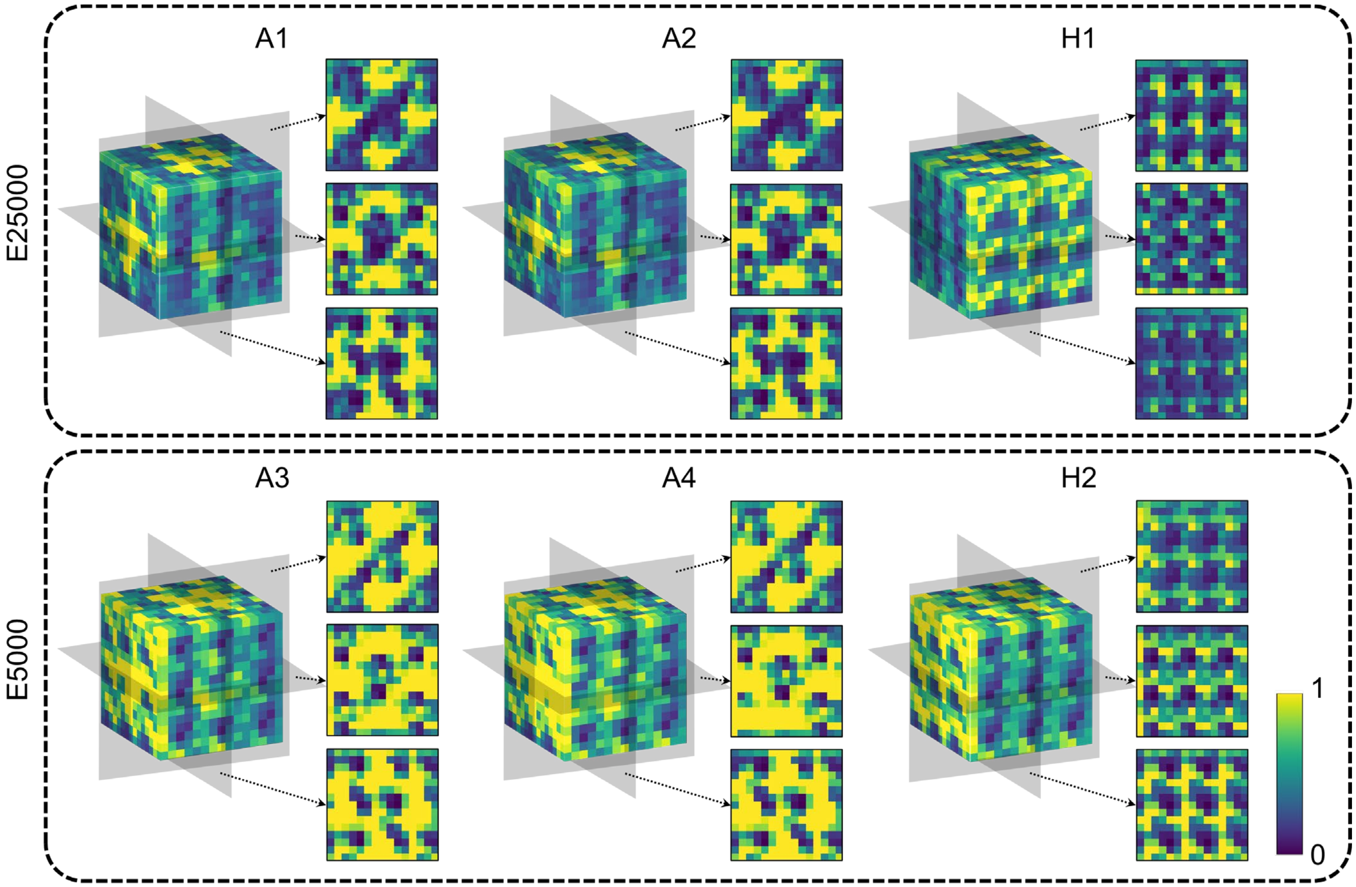

Supplementary Figure 12: Regression activation map of the Ti cubic scaffolds.

The *x-z*, *x-y*, and *y-z* cross-sections of the regression activation map (RAM) of machine learning (ML) designs (A1-A4) and uniform designs (H1 and H2).

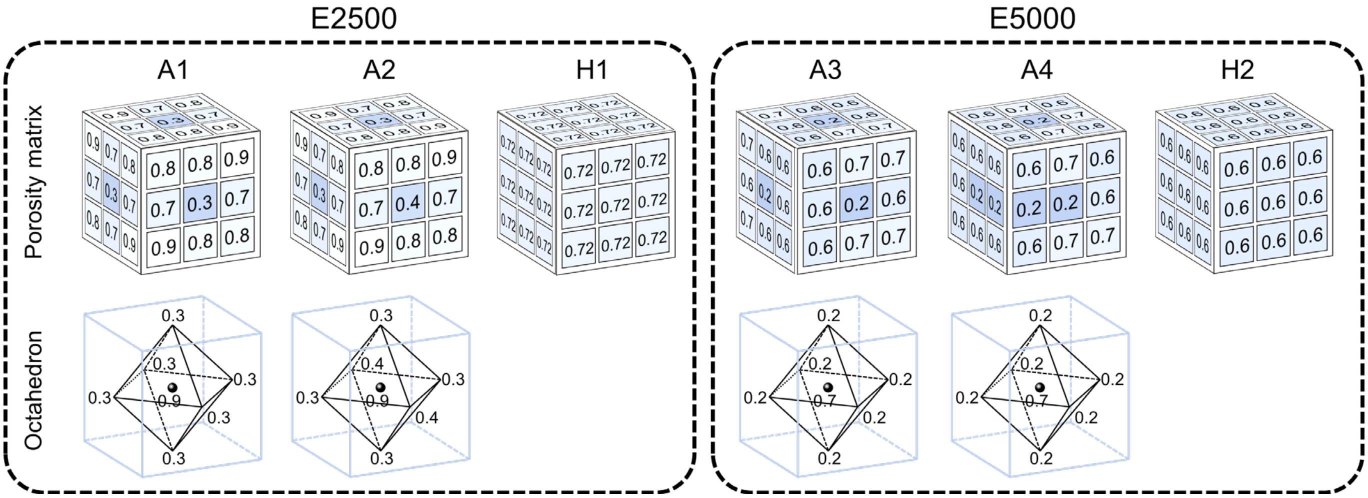

Supplementary Figure 13: The porosity matrices and 'face-centered' lattice structures of the Ti cubic scaffolds.

Porosity matrices of Machine learning (ML) designs (A1-A4) and uniform designs (H1 and H2). The 'face-centered' lattice structures of ML designs (A1-A4).

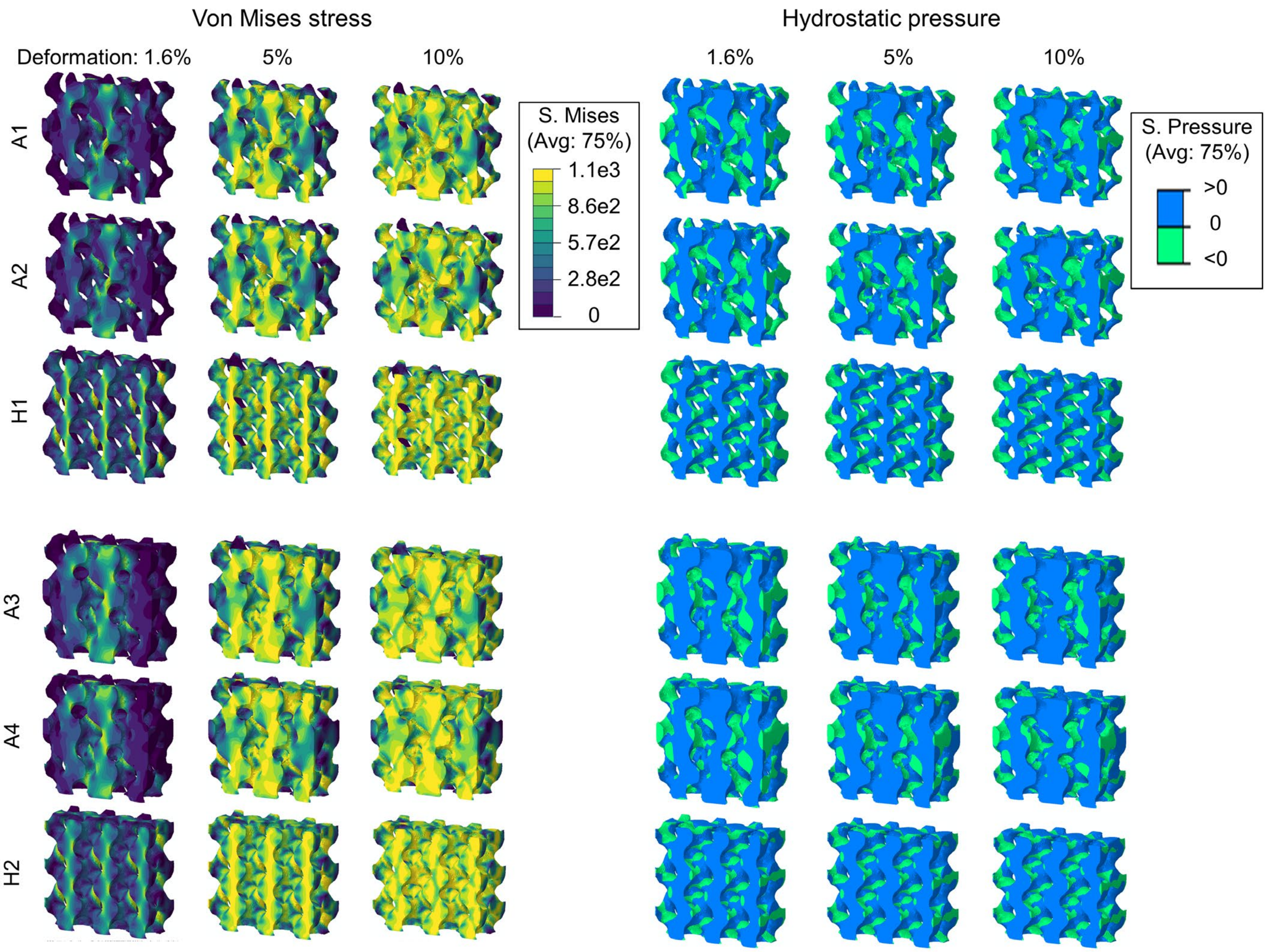

Supplementary Figure 14: FEM analysis of the Ti cubic scaffolds.

Numerical compression analysis of von Mises stress and hydrostatic pressure under 1.6%, 5%, and 10% deformation. Cross-sectional views of the machine learning (ML) designs (A1-A4) and uniform designs (H1 and H2) are plotted. It is noteworthy that current finite element method (FEM) software and ML algorithm work under different operating systems (Windows and Linux, respectively). Hence the data transfer is done manually. To achieve a more automated learning protocol, we believe one of the next steps is to develop a data transfer protocol that enables data communication between different operating systems.

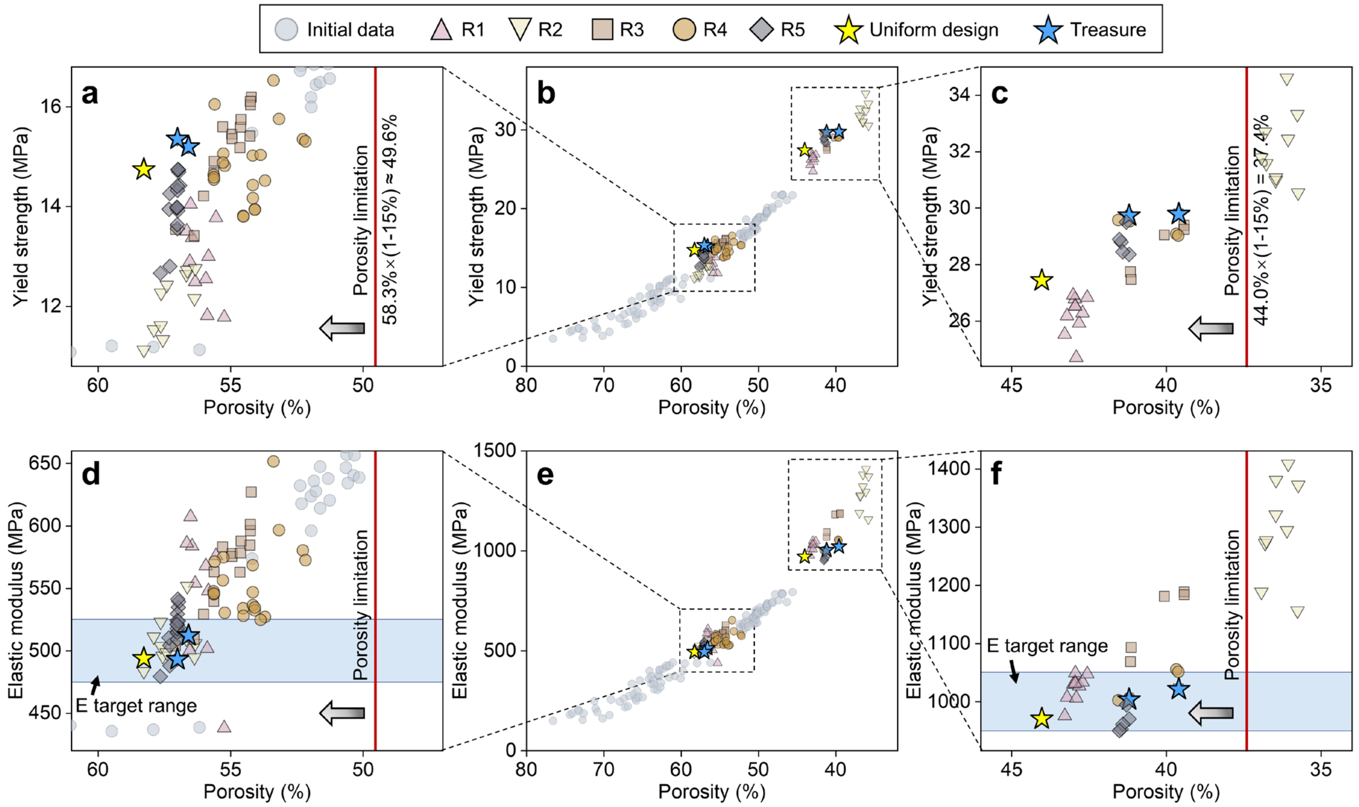

Supplementary Figure 15: The overall data distribution of Zn finite element simulation results.

a, **b**, **c** The porosity-yield strength (Y) plot. The red line indicates the porosity limitation. **d**, **e**, **f** The overall data distribution in terms of the porosity-elastic modulus (E) plot. Initial data points are marked as light blue dots. Five rounds of active learning data points are represented by various other colored symbols (R1-R5). Uniform design and GAD-MALL treasure points are marked as golden and blue stars, respectively, for easier comparison. The blue shaded area indicates the target range of E (i.e., target E value ± 5%). Source data are provided as a Source Data file.

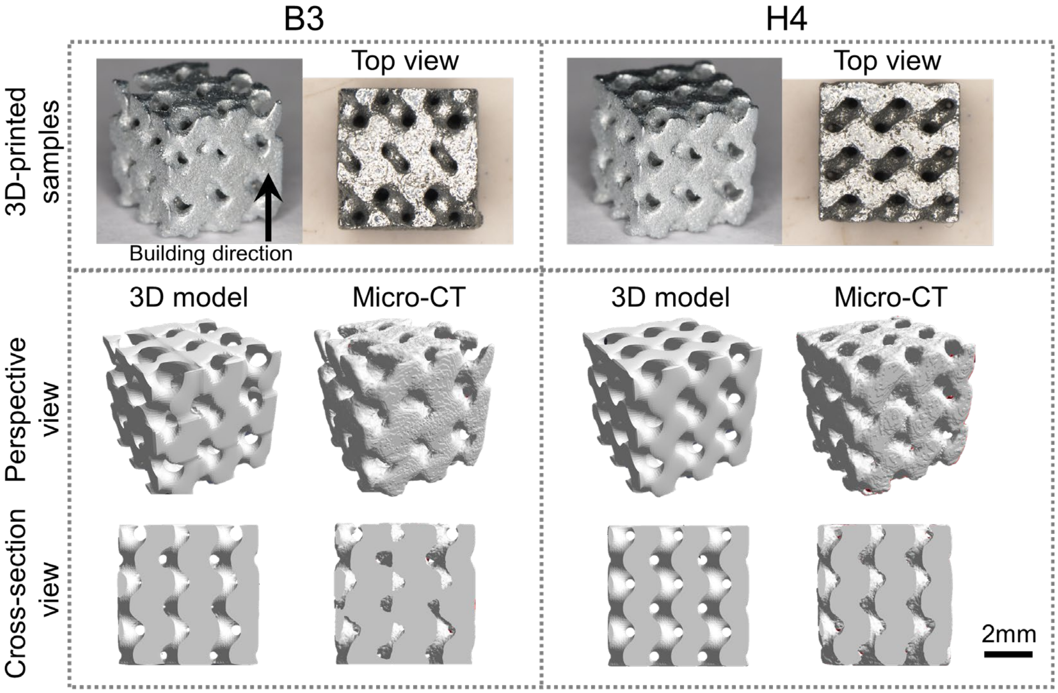

Supplementary Figure 16: Characterization of the Zn cubic scaffolds.

Pictures, Micro-computed tomography (Micro-CT) and 3D models of the machine learning (ML) design (B3) and uniform design (H4).

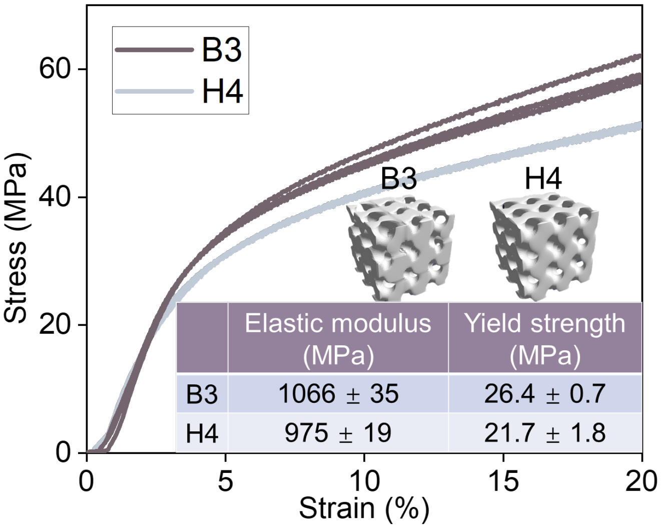

Supplementary Figure 17: Experimental strain–stress curves of the Zn cubic scaffolds.

Machine learning (ML) design (B3) and uniform design (H4). Three replicates were tested to ensure reproducibility. In each figure, all stress-strain curves are adjusted in the x-axis direction to make them overlap. Source data are provided as a Source Data file.

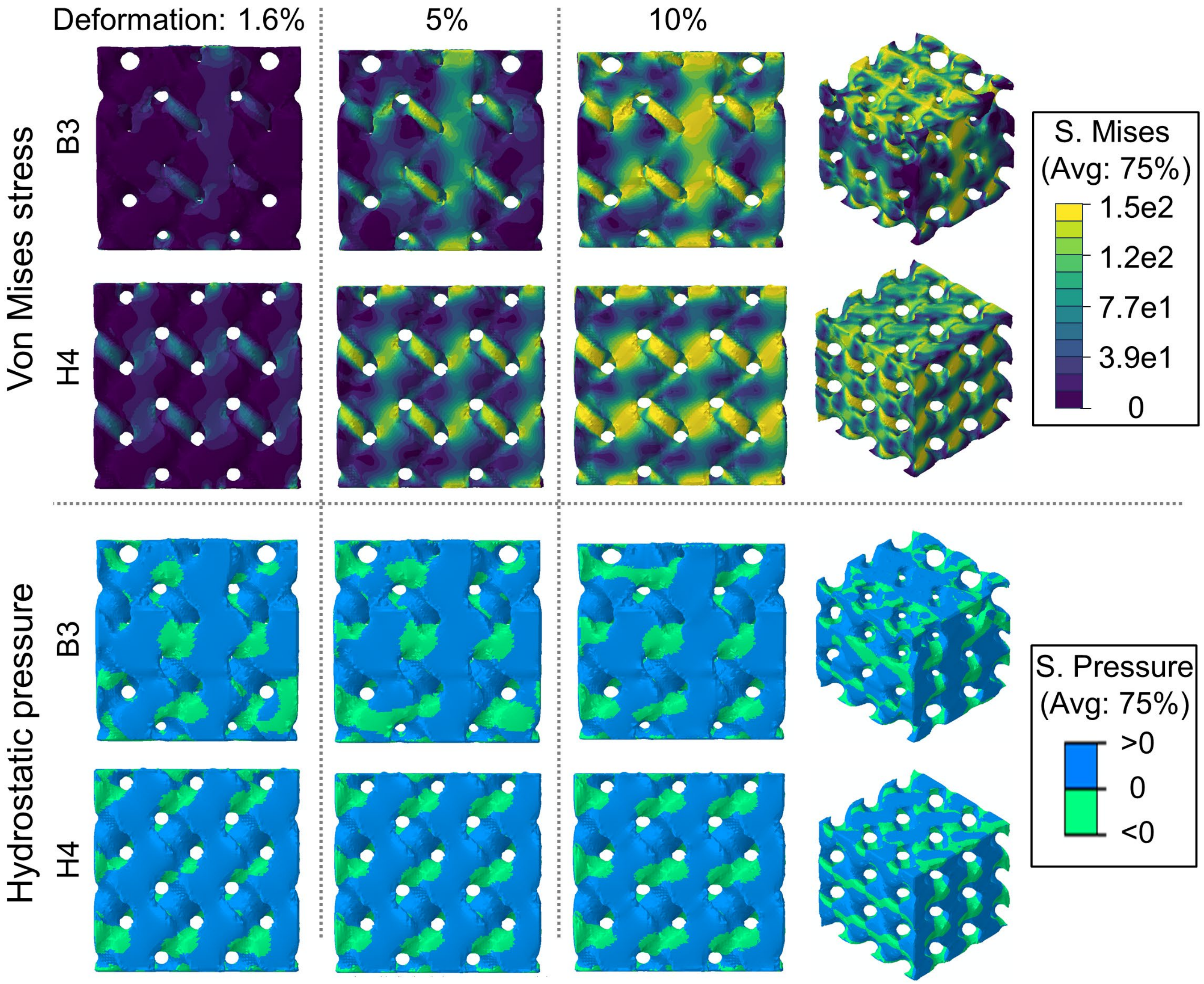

Supplementary Figure 18: FEM analysis of the Zn cubic scaffolds.

Numerical compression analysis of the von Mises stress and hydrostatic pressure under 1.6%, 5%, and 10% deformation. The front and orthographic views of machine learning (ML) design (B3) and uniform design (H4) are plotted.

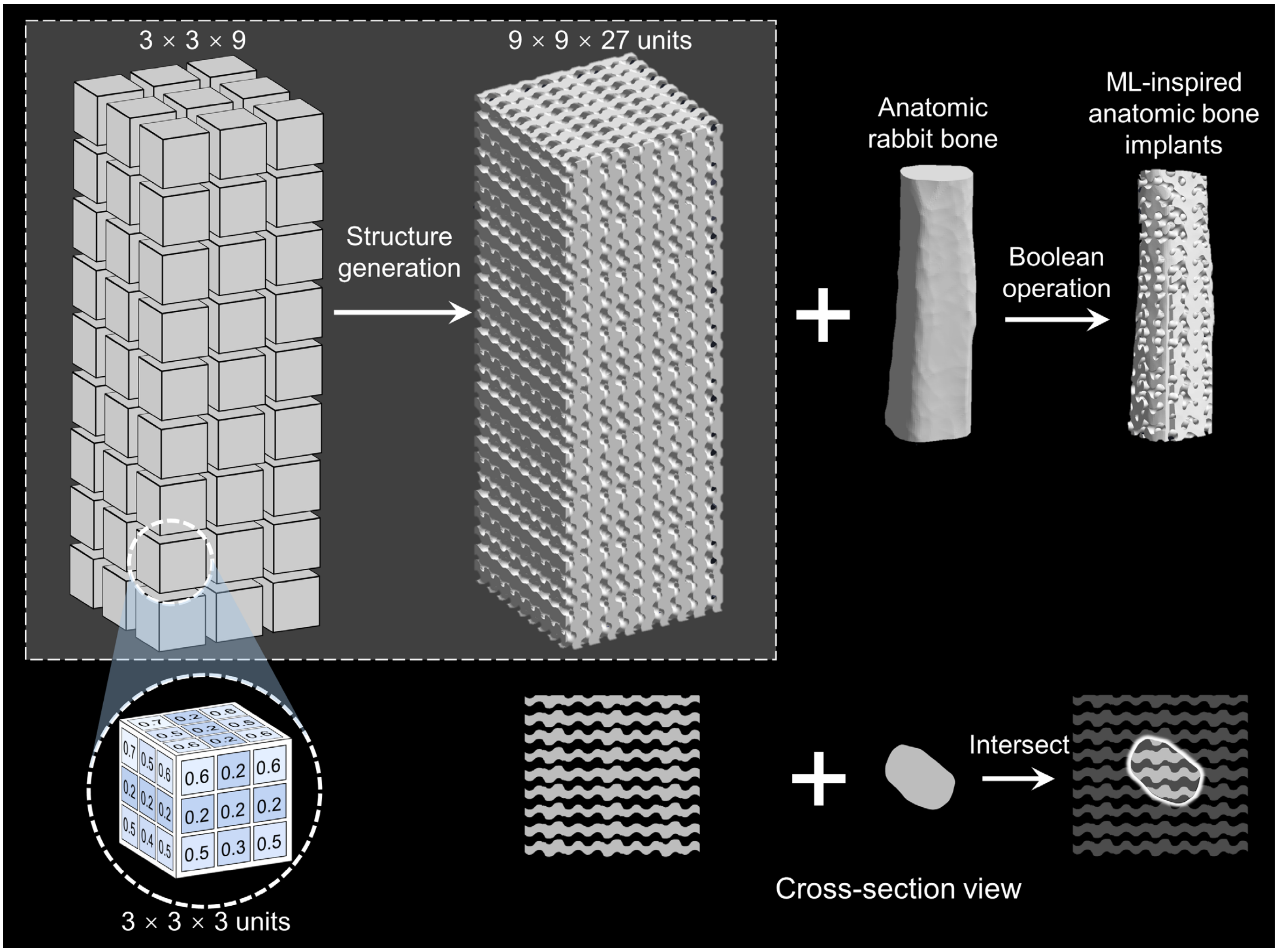

Supplementary Figure 19: Pipeline for irregular-shaped scaffold design.

We used the 3 × 3 × 9 basic structure as the starting point, with each subunit representing the machine learning (ML)-designed cubic scaffold. Overall, the whole structure consisted of 9 × 9 × 27 gyroid units. The scaffold structure was then caved out of the raw materials with the shape matching that of the bones.

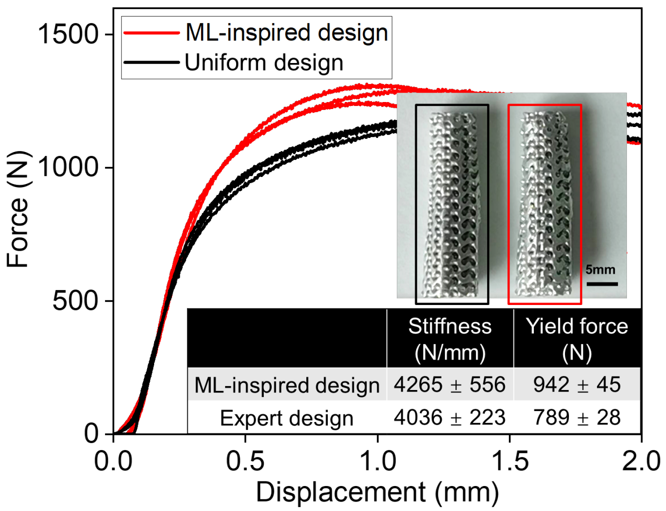

Supplementary Figure 20: Experimental displacement-force curves of bone implants.

The designed bone implant was manufactured, and the experimental compression test was performed. Three replicates were tested to ensure reproducibility. In each figure, all stress-strain curves are adjusted in the x-axis direction to make them overlap. Source data are provided as a Source Data file.

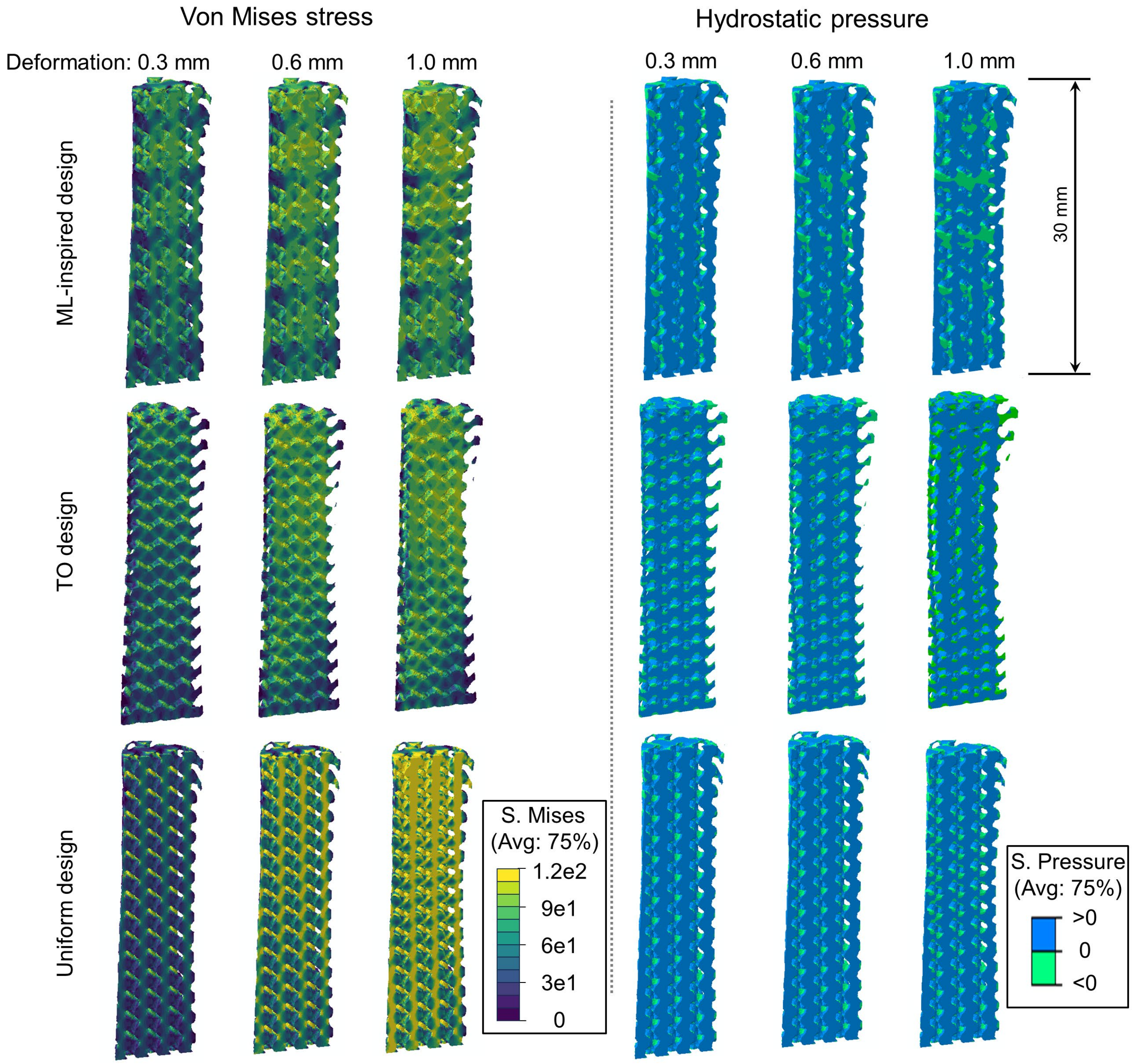

Supplementary Figure 21: FEM analysis of the anatomic bone implants.

Numerical compression analysis of von Mises stress and hydrostatic pressure under 0.3, 0.6, 1.0 mm deformation. The cross-section views of uniform, topology optimization (TO), and machine learning (ML)-inspired designs are plotted.

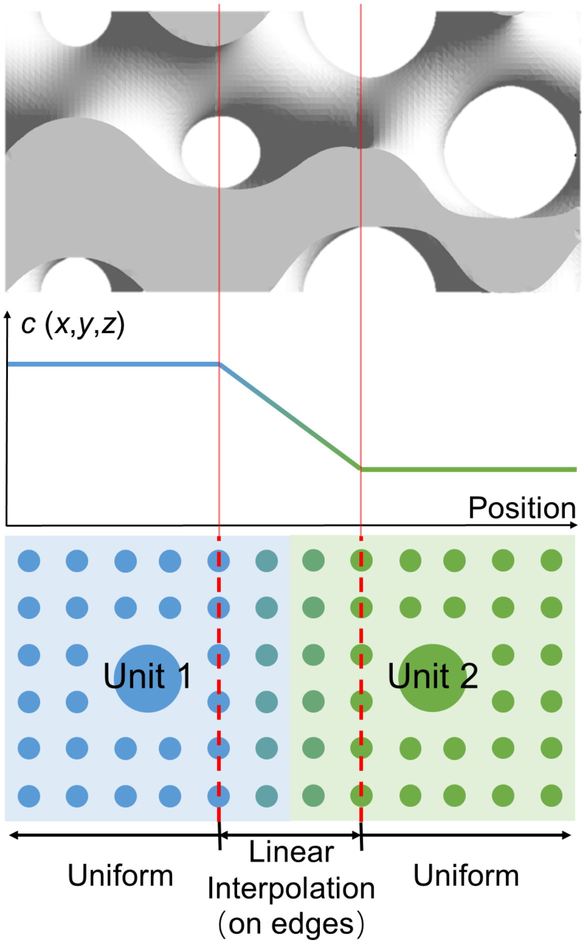

Supplementary Figure 22: The schematic for the smooth transition between units with different porosities.

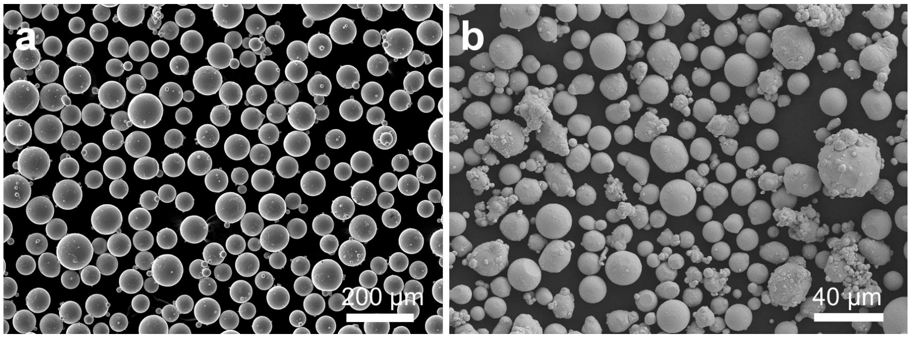

Supplementary Figure 23: Powder observation.
a Ti powder. **b** Zn powder.

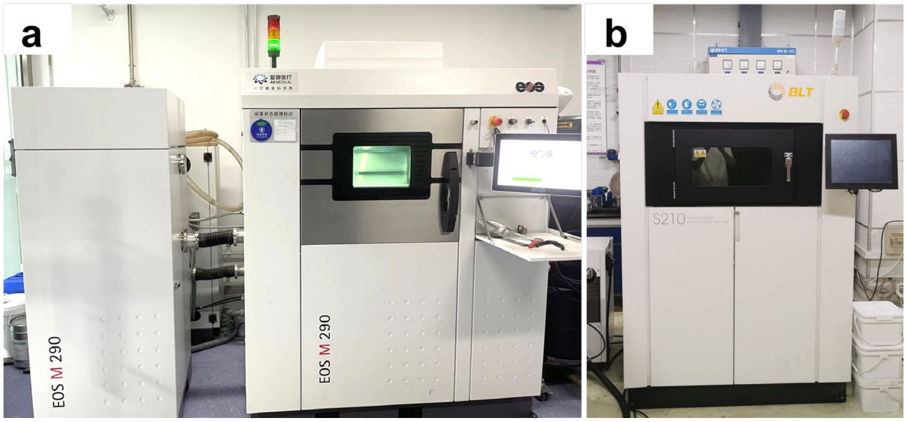

Supplementary Figure 24: Laser powder bed fusion machines of the Ti and Zn cubic scaffolds.

a The EOS M290 machine is used for additively manufacturing the Ti cubic scaffolds. **b** The BLT S210 machine is used for manufacturing the Zn cubic scaffolds.

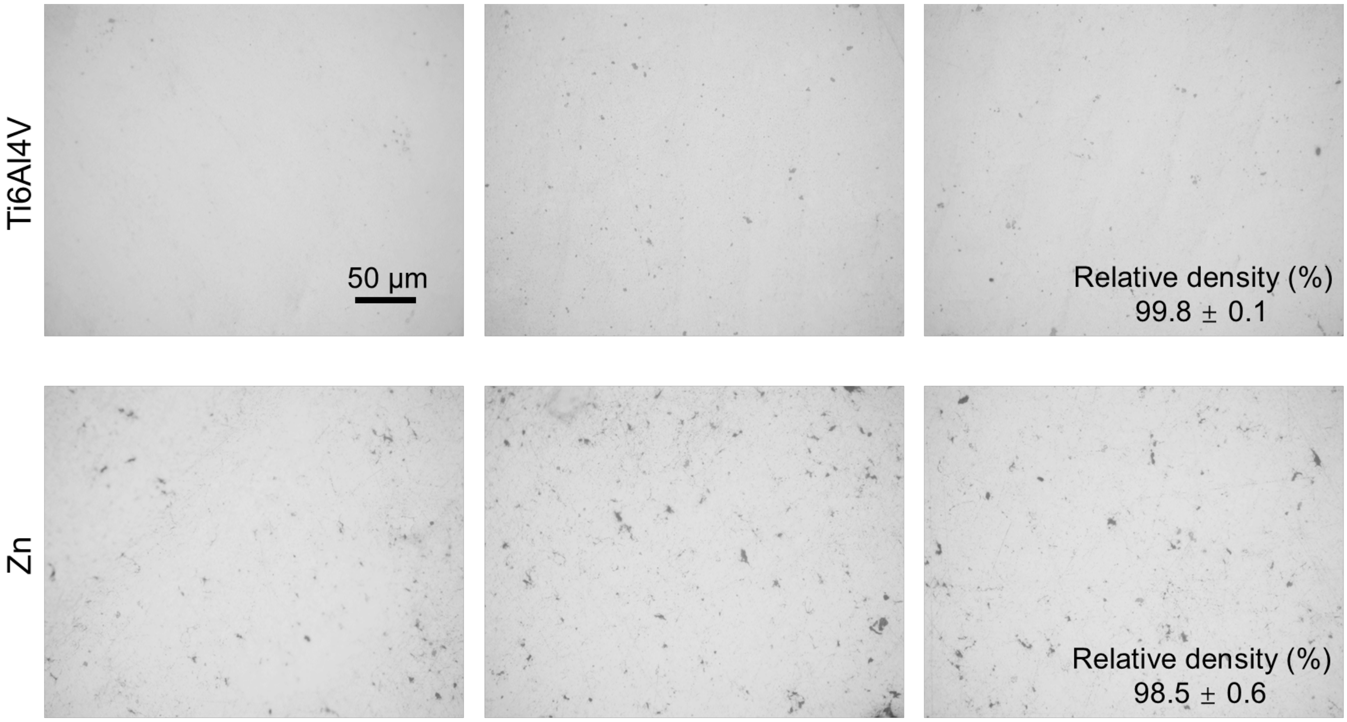

Supplementary Figure 25: Cross-section images of the Ti and Zn cubic scaffolds.

These pictures were used to calculate the relative density of composing struts in cubic scaffolds. Source data are provided as a Source Data file.

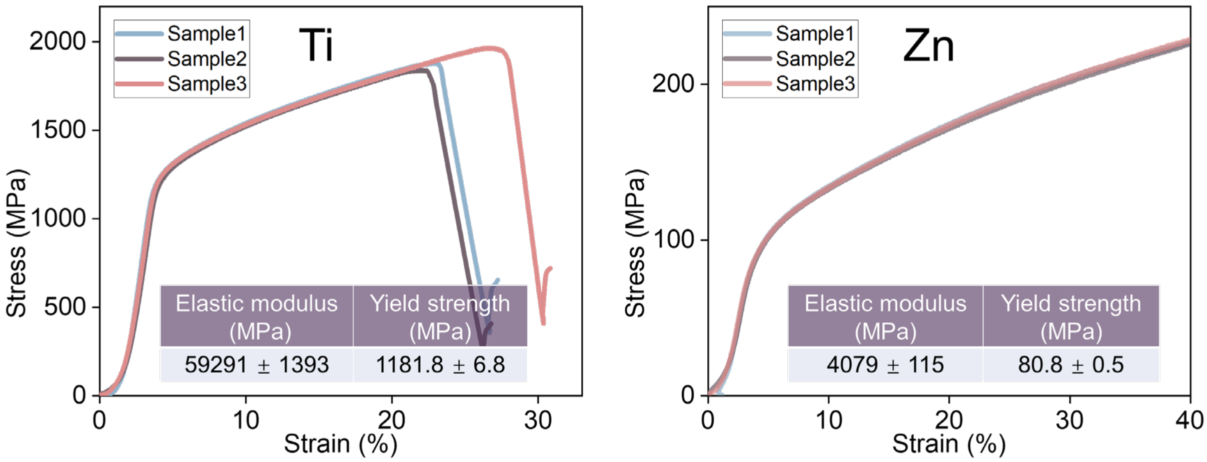

Supplementary Figure 26: Experimental strain–stress curves of solid Ti and Zn.

These curves were used as benchmarks for the FEM parameter calibrations. In each figure, all stress-strain curves are adjusted in the x-axis direction to make them overlap. Source data are provided as a Source Data file.

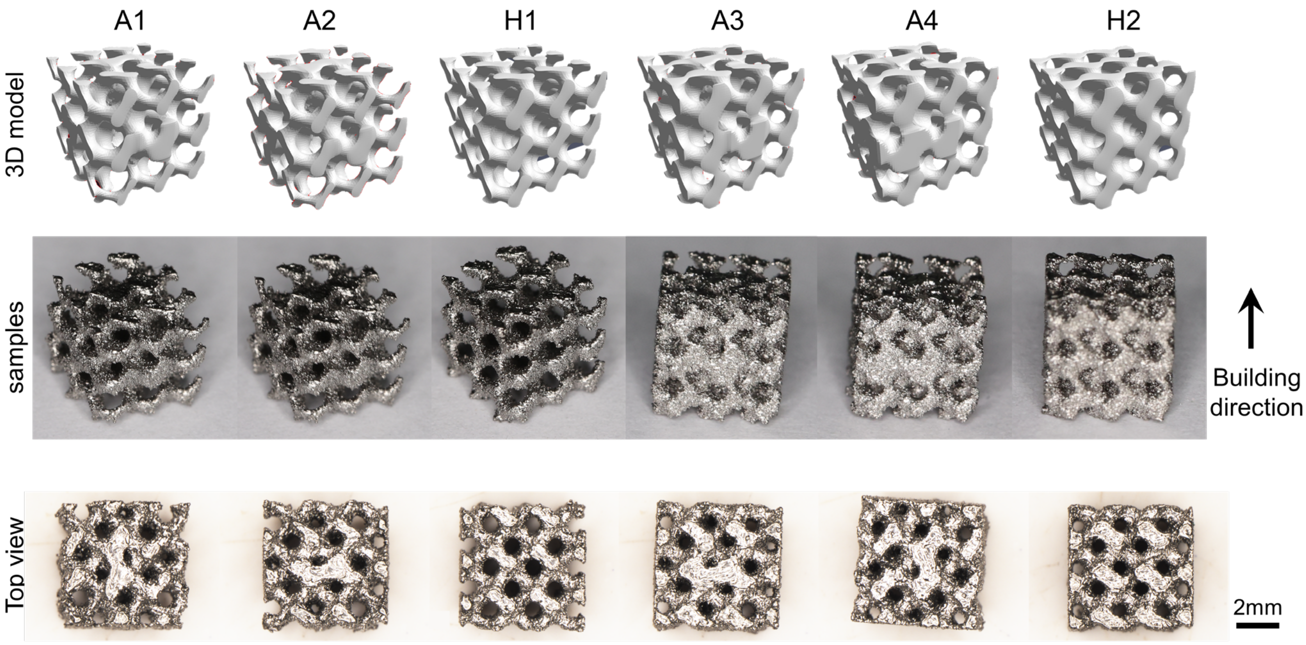

Supplementary Figure 27: The camera pictured Ti cubic scaffolds.

The 3D models, perspective views, and top views of machine learning (ML) designs (A1-A4) and uniform designs (H1 and H2).

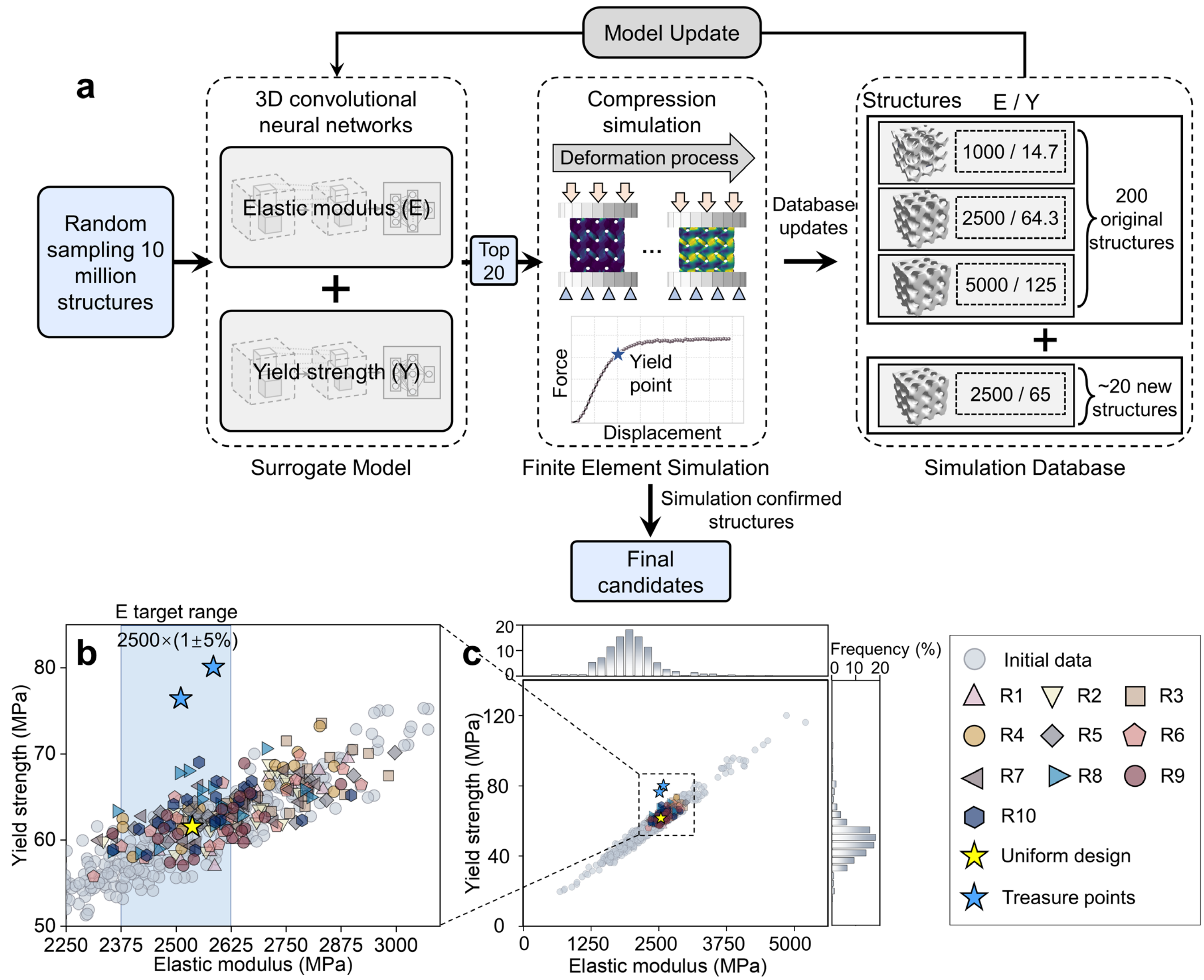

Supplementary Figure 28: Random-search-based active learning.

a Overview of random-search-based active learning. **b**, **c** The elastic modulus (E) – yield strength (Y) distribution of finite element simulation results. Initial data points are marked as light blue dots. Ten rounds of active learning data points are represented by various other colored symbols (R1-R10). Uniform design and GAD-MALL treasure points are marked as golden and blue stars, respectively, for easier comparison. The blue shaded area indicates the target range of E (i.e., target E value ± 5%). Source data are provided as a Source Data file.

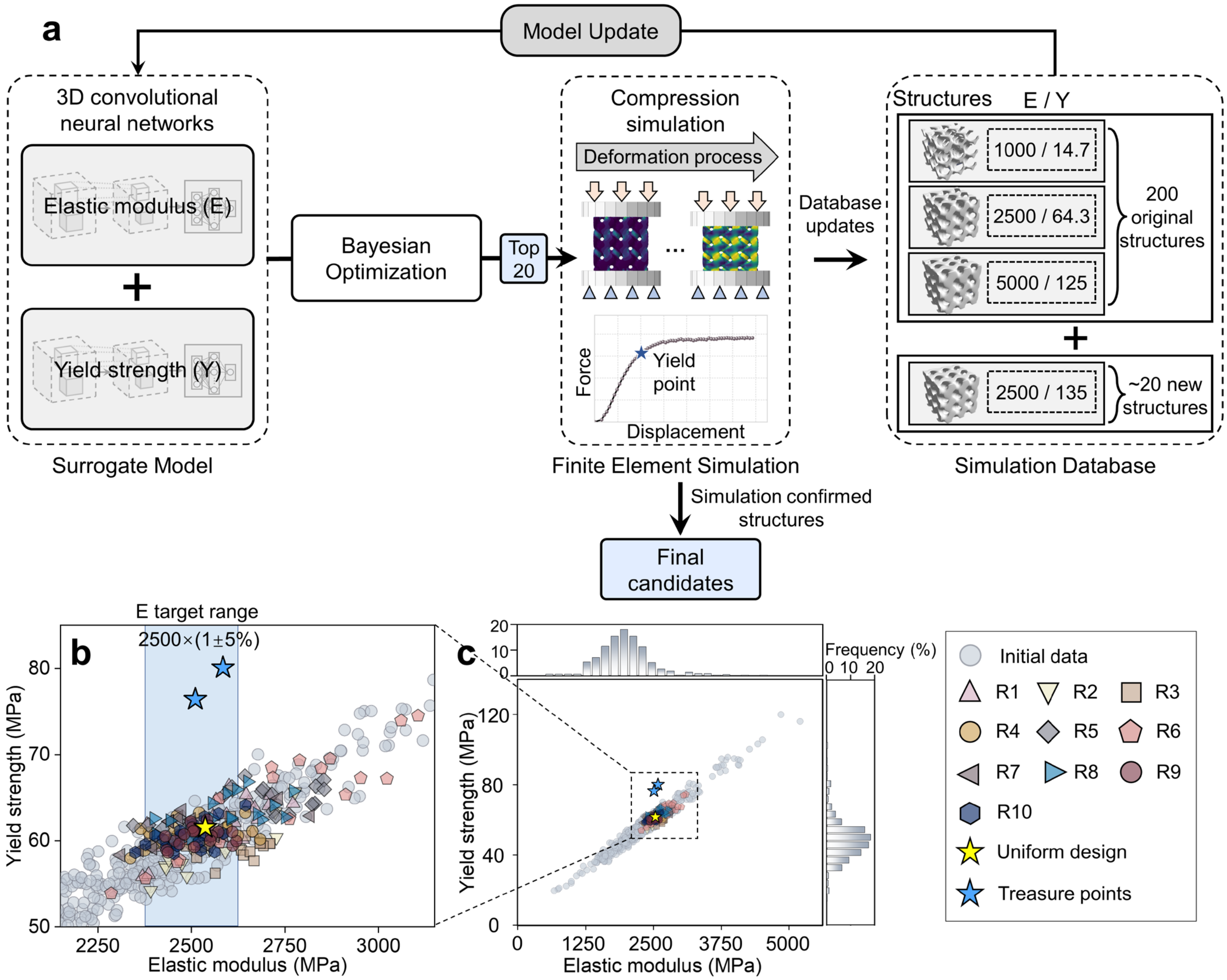

Supplementary Figure 29: Bayesian-optimization-based active learning.

a Overview of Bayesian-optimization-based active learning. **b**, **c** The elastic modulus (E) – yield strength (Y) distribution of finite element simulation results. Initial data points are marked as light blue dots. Ten rounds of active learning data points are represented by various other colored symbols (R1-R10). Uniform design and GAD-MALL treasure points are marked as golden and blue stars, respectively, for easier comparison. The blue shaded area indicates the target range of E (i.e., target E value ± 5%). Source data are provided as a Source Data file.

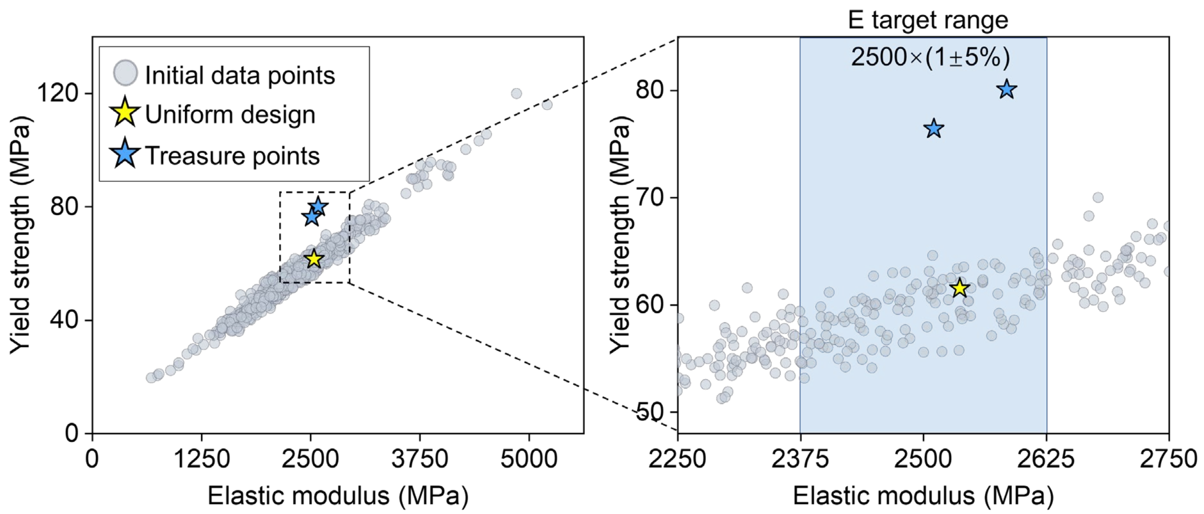

Supplementary Figure 30: Finite element simulation results comparison of newly prepared initial dataset. Initial data points are marked as light blue dots. Uniform design and GAD-MALL treasure points are marked as golden and blue stars for better comparisons. The blue shaded area indicates the target range of E (i.e., target E value ± 5%). Source data are provided as a Source Data file.

Supplementary Tables

Supplementary Table 1: The Ti cubic scaffolds - Mean and standard deviation of the elastic modulus and yield strength at each learning iteration.

Source data are provided as a Source Data file.

		E2500 Task			E5000 Task		
		Elastic modulus (MPa)	Yield strength (MPa)	Porosity (%)	Elastic modulus (MPa)	Yield strength (MPa)	Porosity (%)
Iteration	1	2311 ± 73	57.7 ± 3.3	70.4 ± 2.6	6226 ± 408	139.7 ± 11.8	56.9 ± 1.9
	2	2684 ± 69	62.6 ± 3.1	67.8 ± 2.4	5789 ± 142	136.6 ± 5.3	56.6 ± 1.3
	3	2423 ± 59	65.1 ± 5.3	69.2 ± 2.5	5526 ± 122	124.8 ± 6.8	59.1 ± 0.9
	4	2570 ± 47	66.6 ± 3.9	70.9 ± 1.2	5072 ± 147	119.1 ± 5.8	59.5 ± 0.7
	5	2686 ± 156	69.4 ± 5.2	66.8 ± 3.7	5271 ± 81	129.4 ± 9.8	57.9 ± 2.0
	6	2566 ± 47	70.0 ± 1.7	69.6 ± 1.3	5059 ± 128	136.0 ± 5.9	56.8 ± 1.7

Supplementary Table 2: Experimental results of ML design and uniform design Ti cubic scaffolds.

A1, A2, A3 and A4 represent the best candidates of machine learning (ML) designs for the E2500 and E5000 tasks, respectively. Uniform designs H1 and H2 are the reference scaffolds of E2500 and E5000. Source data are provided as a Source Data file.

	A1	A2	H1	A3	A4	H2
Elastic modulus (MPa)	2527 ± 87	2649 ± 195	2627 ± 154	5169 ± 422	4947 ± 450	4903 ± 303
Yield strength (MPa)	74.8 ± 2.2	73.4 ± 1.9	60.0 ± 1.1	147.6 ± 4.2	147.2 ± 2.3	119.8 ± 8.6
Porosity (%)	69.3 ± 0.3	69.1 ± 0.1	73.0 ± 0.2	57.5 ± 0.3	55.6 ± 0.1	60.8 ± 0.3

Supplementary Table 3: The Zn cubic scaffolds - Mean and standard deviation of the elastic modulus and yield strength at each learning iteration.

Source data are provided as a Source Data file.

		E500 Task			E1000 Task		
		Elastic modulus (MPa)	Yield strength (MPa)	Porosity (%)	Elastic modulus (MPa)	Yield strength (MPa)	Porosity (%)
Iteration	1	546 ± 52	12.9 ± 0.8	56.1 ± 0.5	1024 ± 22	26.2 ± 0.7	42.9 ± 0.2
	2	508 ± 19	12.1 ± 0.6	57.2 ± 0.7	1297 ± 85	32.1 ± 1.3	36.3 ± 0.4
	3	568 ± 34	15.2 ± 0.9	55.1 ± 0.9	1123 ± 71	28.8 ± 0.9	40.1 ± 1.3
	4	555 ± 31	14.8 ± 0.7	54.3 ± 1.1	1024 ± 27	29.4 ± 0.3	40.6 ± 0.9
	5	515 ± 16	14.2 ± 0.7	57.1 ± 0.2	965 ± 18	28.8 ± 0.5	41.4 ± 0.1

Supplementary Table 4: Experimental results of ML design and uniform design Zn cubic scaffolds.

B1, B2, B3 and B4 represent the best candidates of machine learning (ML) designs for E500 and E1000 tasks, respectively. H3 and H4 are the reference scaffolds (uniform porosity) of E500 and E1000. The B3 scaffold shows better performance than the gold criteria H4 scaffold. Source data are provided as a Source Data file.

	B1	B2	H3	B3	B4	H4
Elastic modulus (MPa)	484 ± 17	510 ± 28	510 ± 28	1066 ± 35	1012 ± 22	975 ± 19
Yield strength (MPa)	13.0 ± 0.6	12.7 ± 0.3	12.9 ± 0.5	26.4 ± 0.7	21.8 ± 0.7	21.7 ± 1.8
Porosity (%)	56.5 ± 0.5	56.8 ± 0.2	55.8 ± 0.1	35.4 ± 0.2	38.6 ± 0.1	39.3 ± 0.4

Supplementary Table 5: The actual porosity of scaffolds from Micro-CT characterization.

	A1	H1	A4	H2	B3	H4
3D Model porosity (%)	68.9	72.0	55.7	60.7	39.6	44.0
Real porosity (%)	69.2	72.2	55.4	61.1	34.9	39.2

Supplementary Table 6: Comparison of GAD-MALL, random-search-based active learning and Bayesian-optimization-based active learning.

Method	Initial data points	Iteration	Number of sampling points	Time cost for ML	Time per point	Strength increase
GAD-MALL	95	6	10 million	2 h	0.0007 s	30.1%
Random Search	521	10	10 million	2 h	0.0007 s	12.1%
Bayesian Optimization	521	10	~ 4000	6 h	5.4 s	7.6%

Supplementary Methods

Baseline comparison

To show that Generative Architecture Design - Multi-objective Active Learning Loop (GAD-MALL) outperforms the other baselines, we provided additional machine learning (ML) experiments and compared GAD-MALL with other state-of-the-art approaches (random search in Supplementary Fig. 28 and Bayesian optimization in Supplementary Fig. 29). It turned out that these methods could not find the structures with better mechanical strength, even with more data and more iterative rounds. We prepared 521 initial data points (about 4 times more than GAD-MALL initial dataset (95 data points), Supplementary Fig. 30) and the target is to optimize the yield strength at E = 2.5 GPa. In both cases, we used the 3D convolutional neural network (3D-CNN) as the surrogate model. Technical details for random search: we randomly sampled 10 million structures at each iteration and coupled it to the 3D-CNN to form an active learning loop. More information is available in Supplementary Table 6. The code of the protocol is available at https://github.com/Bop2000/GAD-MALL and https://doi.org/10.5281/zenodo.8281073.

To further test the robustness and applicability of GAD-MALL, we performed the optimization tasks on the well-known Rosenbrock function, which is a classic example of a non-linear, high-dimensional function with known global optimal solution and frequently used to test optimization algorithms[2]. Here, we adopted a 20-dimensional form of the Rosenbrock function:

$$f(\mathrm{X}) = \sum_{i=1}^{N-1}\left[(a-x_i)^2 + b(x_{i+1} - cx_i^2)^2\right] \quad \text{where } \mathrm{X}=(x_1,\ ...,\ x_N) \in \mathbb{R}^N$$

For small N, the polynomials can be determined precisely, and Sturm's theorem can be employed to ascertain the number of real roots. However, this method becomes ineffective for larger N due to the magnitude of the coefficients. By setting a, b, c, and N equal to 0.2, 2, 5, and 20 respectively, there is exactly one global minimum of 0 at (0.2, 0.2, …, 0.2) and numerous local minima, such as (0, 0, …, 0).

In this study, we compared GAD-MALL with random search and Bayesian optimization by setting x_i in the range (0, 0.5) with a step size of 0.05. The Rosenbrock was reversed with a minus sign so that it becomes a global maximum search problem. We start with 20000 initial data points for the training of neural network, and the active learning strategy is to update the dataset the CNN-predicted top 500 to the dataset at each round. The result is shown in Supplementary Fig. 9, as one can see the GAD-MALL outperforms other two baseline approach, reaching the global maximum at 11th round. Whereas random and Bayesian search quickly get trapped in the local minimum, showing little improvement even after 20 rounds.

Supplementary References

1. Li, D. *et al.* Optimal design and modeling of gyroid-based functionally graded cellular structures for additive manufacturing. *Computer-Aided Design* **104**, 87–99 (2018).
2. Rosenbrock, H. An automatic method for finding the greatest or least value of a function. *Comput J* **3**, 175–184 (1960).
